# Subducting volcaniclastic-rich upper crust supplies fluids for shallow megathrust and slow slip

**DOI:** 10.1126/sciadv.adh0150

**Published:** 2023-08-16

**Authors:** Andrew C. Gase, Nathan L. Bangs, Demian M. Saffer, Shuoshuo Han, Peter K. Miller, Rebecca E. Bell, Ryuta Arai, Stuart A. Henrys, Shuichi Kodaira, Richard Davy, Laura Frahm, Daniel H. N. Barker

**Affiliations:** ^1^ Institute for Geophysics, Jackson School of Geosciences, University of Texas at Austin, Austin, TX, USA.; ^2^ Department of Earth Science and Engineering, Imperial College London, London, UK.; ^3^ Research Institute for Marine Geodynamics, Japan Agency for Marine-Earth Science and Technology, Yokohama, Japan.; ^4^ GNS Science, Lower Hutt, New Zealand.

## Abstract

Recurring slow slip along near-trench megathrust faults occurs at many subduction zones, but for unknown reasons, this process is not universal. Fluid overpressures are implicated in encouraging slow slip; however, links between slow slip, fluid content, and hydrogeology remain poorly known in natural systems. Three-dimensional seismic imaging and ocean drilling at the Hikurangi margin reveal a widespread and previously unknown fluid reservoir within the extensively hydrated (up to 47 vol % H_2_O) volcanic upper crust of the subducting Hikurangi Plateau large igneous province. This ~1.5 km thick volcaniclastic upper crust readily dewaters with subduction but retains half of its fluid content upon reaching regions with well-characterized slow slip. We suggest that volcaniclastic-rich upper crust at volcanic plateaus and seamounts is a major source of water that contributes to the fluid budget in subduction zones and may drive fluid overpressures along the megathrust that give rise to frequent shallow slow slip.

## Teaser

Clay rich volcanic materials supply voluminous water to a subduction zone that hosts recurring slow slip.

## INTRODUCTION

Subducting oceanic lithosphere and marine sediments carry large amounts of pore- and mineral-bound water that influence the mechanical behavior of megathrust faults (*1*) and chemical cycles to the deep Earth (*2*, *3*). Upon subduction at ocean trenches, increasing stress loads the rock framework, generating excess fluid pressures that drive fluid flow through the forearc (*4*, *5*). As temperatures warm to ~60° to 150°C, low-temperature metamorphic reactions begin to release mineral-bound water from clays and opal into the outer forearc (*6*–*8*). Pore collapse reduces formation permeability (*9*), enhancing tendencies for overpressure generation (*10*) and increasingly channelized drainage pathways at greater depths (*5*).

Where fluids are released has important consequences for crustal strength, deformation, and earthquake processes within the subduction system. Subducting lithosphere that is hydrated by outer rise bend-faulting and fracture zones (*11*–*13*) is suggested as a source of fluids that contribute to intermediate-depth intraplate earthquakes (*14*, *15*) and influence the chemical composition of arc magmas (*16*, *17*). Shallow slow earthquakes, including slow slip events (*18*, *19*), tectonic tremor (*20*), and low-frequency earthquakes (*21*), can occur when faults with slightly rate-weakening friction experience low-effective normal stresses induced by fluid overpressures (*22*–*24*); the major sources of these fluids are often assumed to be compacting and dehydrating marine sediments (*1*, *25*, *26*). However, well constrained fluid budgets are limited to the closest few kilometers from the trench and their direct links to slip processes are not always clear. Correlations between slow slip, rough subducting seafloor (*27*), and heterogeneous fault zones (*28*, *29*) indicate that material mixing between subducting volcanic basement and sediments may also have important controls on megathrust slip modes. Some regions with well-observed shallow slow slip, such as the northern Hikurangi margin (*30*, *31*), the Mid-America trench (*21*, *32*), and the Ryukyu trench (*33*, *34*), have relatively thin subducting sediment units (<200 m) that would yield a limited source of fluids and do not buffer the megathrust from subducting topography.

Whether subducting crust and seamounts influence shallow megathrust hydrogeology is an outstanding question with few observations. The close correspondence between rough seafloor and slow slip has motivated hypotheses that seamounts transport large quantities of water to the shallow megathrust, either indirectly by shadowing trailing sediments from collisional stresses (*35*, *36*) or directly within the seamounts’ thick extrusive and altered volcanic layers (*37*, *38*). Vigorous hydrothermal circulation is observed within unsubducted seamounts that are not buried by sediments (*39*). While hydrothermal circulation within basaltic oceanic crust is maintained during subduction (*40*), little evidence exists to suggest widespread mobilization of basement pore fluids in response to seamount subduction or substantial flux across the sediment-basement interface (*8*), which would be required to influence megathrust hydrogeology. Some studies of pore-water geochemistry do suggest upward flow from deep-seated metasomatic fluid sources within subducting oceanic lithosphere (*3*, *41*, *42*). However, subducting sediments are a more likely source of fluid for the shallow megathrust than upper basaltic oceanic crust because sediments are more compressible than basaltic crust, sediments often contain hydrous minerals that dewater at low temperatures, and the shallow megathrust is typically surrounded by sediments (*1*, *6*, *43*, *44*).

**Fig. 1.**
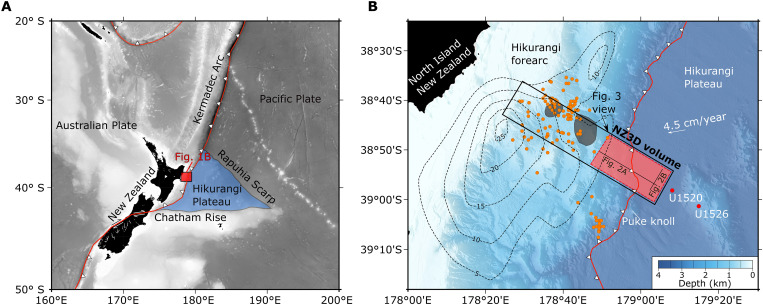
Tectonic setting of the northern Hikurangi margin. (**A**) Regional map showing the extent of the Hikurangi Plateau (blue polygon) and major tectonic plate boundaries (red lines) in the southwest Pacific. (**B**) Bathymetric map showing the deformation front of the Hikurangi margin (red line with white triangles). The Hikurangi Plateau subducts at ~4.5 cm/year (*55*). The area of the entire NZ3D seismic survey is contained within the black rectangle, and the subregion of interest in this study is covered by the red shaded box. Select 2D profiles presented in Fig. 2 are displayed as black lines. IODP Expedition 375 sites U1520 and U1526 (red circles) drilled into volcanic rocks of the Hikurangi Plateau. Subducting seamounts within the NZ3D volume identified by (*36*) are shaded in gray. Dashed black contours are estimated megathrust slip (cm) in a 2014 slow slip event (*18*), and orange circles are associated tectonic tremors (*20*).

The NZ3D seismic experiment was designed to image the source region of slow slip events at New Zealand’s northern Hikurangi margin with resolution that is unprecedented at any subduction zone (Fig. 1). Here, slow slip events are reported every 1 to 2 years along the megathrust between the trench and 20 km depth (*30*). A 15 × 60 km^2^ seismic volume was acquired with four, 6 km long hydrophone streamers and two 3300 in^3^ tuned source arrays of *R/V Langseth*, and 97 short-period ocean bottom seismometers. Following acquisition, we contracted CGG Services (Singapore) Ltd. to conduct advanced noise reduction, three-dimensional (3D) multiple removal, 3D acoustic full-waveform inversion and reflection tomography, and 3D prestack depth migration (PSDM) (*36*, *45*–*47*). We report evidence for extensive dewatering within the volcanic upper crust within 15 km of the trench in response to vertical loading. Large quantities of water (~18 to 30 vol % H_2_O) remain within the volcanic upper crust as it subducts into regions of recurring, well-characterized shallow slow slip (Fig. 1).

## RESULTS

### The upper crust fluid reservoir within the Hikurangi Plateau

The Hikurangi Plateau large igneous province (LIP) formed in the Early Cretaceous [~120 to 125 million years (Ma)] during the Greater Ontong Java magmatic event along with the Ontong Java and Manihiki Plateaus (Fig. 1) (*48*). After rifting from the Ontong Java and Manihiki Plateaus, the Hikurangi Plateau underthrust the East Gondwana margin and subduction ceased between ~70 and 105 Ma (*49*, *50*). The Hikurangi Plateau was also modified by a major phase of intraplate volcanism in the Late Cretaceous (67 to 99 Ma) that constructed large seamounts with basal dimensions up to ~70 km (*49*, *51*). Subduction at the Hikurangi margin resumed sometime between the Eocene and Miocene, but the extent and timing of Hikurangi Plateau subduction is poorly constrained (*52*). Kermadec arc lavas south of ~32°S are enriched in fluid mobile trace elements and contain isotopic signatures that suggest subduction and mixing with a buoyant Hikurangi Plateau mélange ~250 km north of the Hikurangi Plateau’s current position (*53*). Within the NZ3D survey, 8 to 11 km thick Hikurangi Plateau crust (*31*, *54*) subducts westward at ~4.5 cm/year (Fig. 1B) (*55*). Recent drilling at the large seamount nearest to NZ3D, Tūranganui Knoll, recovered 198 m of smectite clay-rich volcaniclastic conglomerate with basalt and silty volcanic claystone interbeds along the seamount’s flank, and sampled 40 m of alternating beds of vesicular basalt and volcaniclastic conglomerate near the summit (*29*, *56*, *57*).

**Fig. 2.**
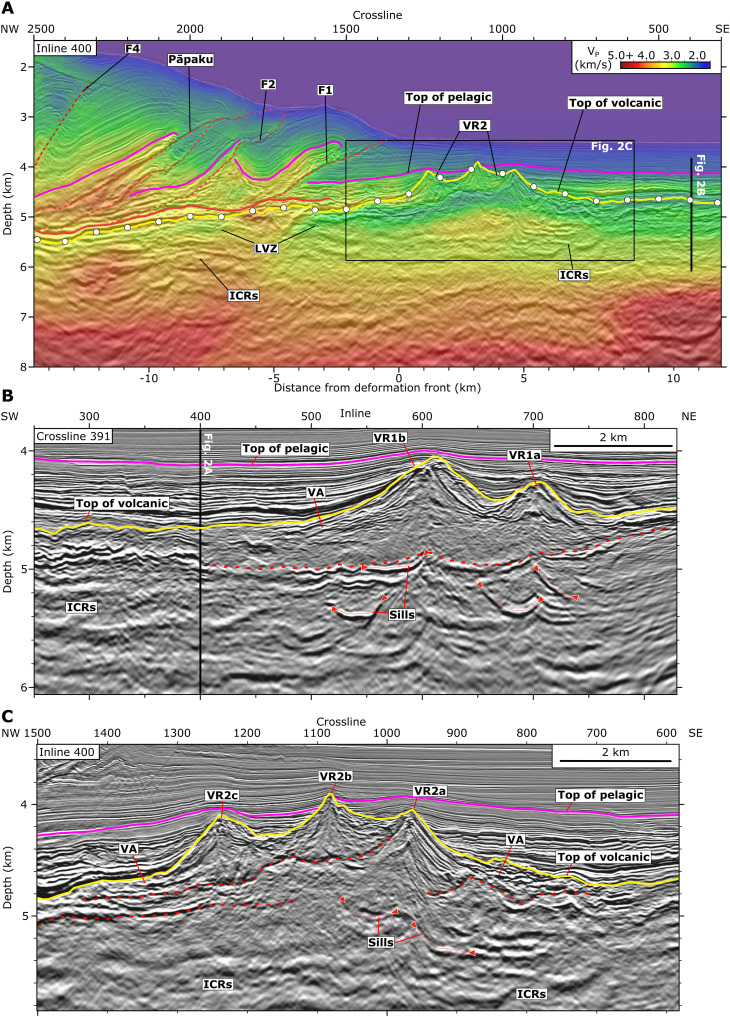
Seismic profiles from the NZ3D reflection and velocity volumes showing volcanic features and faults in the overthrusting plate. (**A**) *P*-wave velocity model and PSDM reflection image containing the Hikurangi Plateau and accretionary prism at inline 400. Yellow line with white circles is the top of volcanic basement [HKB/VB of (*29*, *49*)]. Magenta line is the top of pelagic units. Red solid line highlights the megathrust, and red dashed lines highlight thrust faults that separate accreted thrust sheets in the prism (labeled F1, F2, Pāpaku, and F4). The low-velocity zone (LVZ) in the subducting volcanic upper crust persists to >15 km from the deformation front. Intracrustal reflections (ICRs) are interpreted as extrusive flows and sills within the upper crust. (**B**) Enlarged panel from crossline 391 showing saucer-shaped sills that transgress ICRs below beneath volcanic cones of VR1. The base of VR1 is marked by a dotted red line. Sill tips are annotated as red triangles. VA, volcaniclastic apron. (**C**) Enlarged panel of VR2 in (A). The base of younger cones (VR2b and VR2c) and an older cone (VR2a) is marked by red dotted lines. Vertical exaggeration is 2:1.

**Fig. 3.**
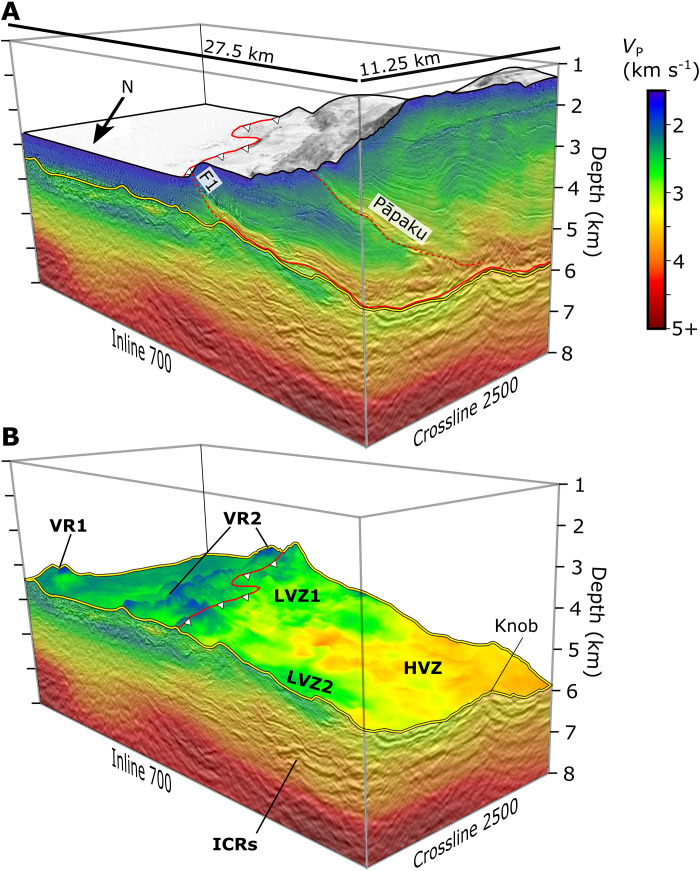
Three-dimensional perspectives of the superimposed NZ3D velocity and reflection volumes. The perspective is facing south-southeast looking up-dip of the subducting Hikurangi Plateau with respect to the subduction direction. The deformation front is marked by the barbed solid red line. (**A**) Volume cut at the seafloor. Red solid and dashed lines are the décollement and major thrust faults. The top of volcanic basement is the solid yellow line. (**B**) Volume cut at the top of volcanic basement. Annotations show the locations of volcanic regions (VR1 and VR2), LVZs (LVZ1 and LVZ2), a high-velocity zone (HVZ), and intracrustal reflectors (ICRs). Vertical exaggeration is 2:1.

We focus on hydrogeologic processes inferred from seismic reflection and *P*-wave velocity data in a subregion of the NZ3D volume spanning 15 km to either side of the trench (Fig. 1). We limit the down-dip extent of our analysis to the region where we can confidently identify the top of the Hikurangi Plateau volcanic upper crust. The Hikurangi Plateau is draped with a 0.4 to 1.2 km thick sedimentary blanket composed of Late Cretaceous siliciclastics, Paleocene-Pleistocene pelagic carbonates, and Pleistocene-present hemipelagic turbidites (*58*) that thins near volcanic cones (Fig. 2). At the deformation front, velocities within the sedimentary cover rise, reflecting consolidation in response to greater stress. The décollement forms beneath the frontal thrust ridge and can be traced landward along a reflective horizon (Fig. 2A). Some thrust sheet hanging walls contain higher velocity materials, presumably pelagic carbonates, that are underlain by lower-velocity footwalls (Figs. 2A and 3A). Subducting calcareous pelagic sediments near the deformation front are ~0.2 km thick or less and are laterally discontinuous such that the décollement sometimes intersects the top of volcanic basement (Figs. 2A and 3A) (*29*, *31*, *36*).

**Fig. 4.**
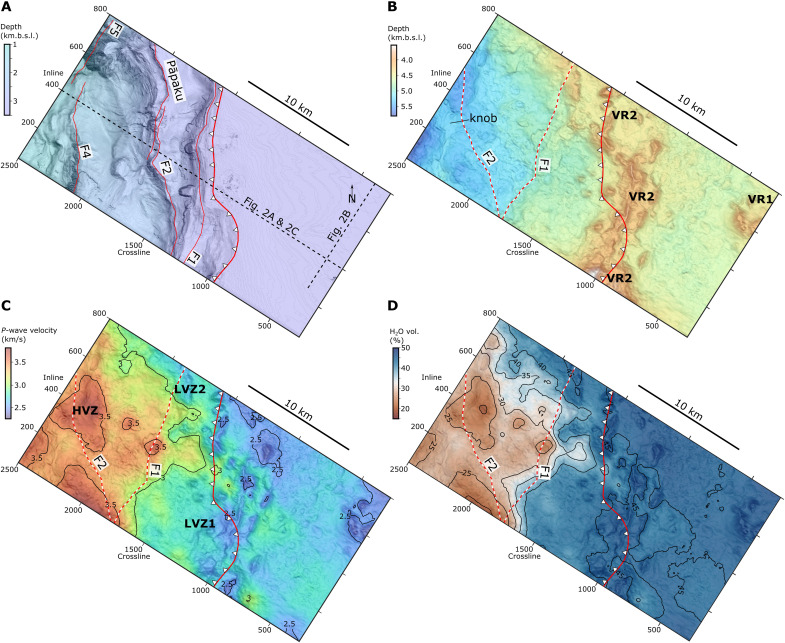
Map perspectives of seismic horizons and seismically derived physical properties within the upper 1 km of the Hikurangi Plateau. (**A**) Seafloor bathymetry from the seafloor seismic reflection with the deformation front (barbed solid red line) and major thrust fault scarps (solid red lines). (**B**) Topography of the top of the Hikurangi Plateau upper crust (HKB/VB). VR1 and VR2, volcanic regions 1 and 2 (see Figs. 2 and 3). The approximate location where major thrust faults intersect the décollement is shown as red and white dashed lines. (**C**) Mean *P*-wave velocity in the upper 1 km of the Hikurangi Plateau. (**D**) Average volumetric water content in the upper 1 km of the Hikurangi Plateau.

The 3D images of the subducting plate help us constrain the geologic nature of the shallow crust and assess its fluid capacity. We identify the top of the Hikurangi Plateau as an unconformity [i.e., Hikurangi Basement/Volcanic Basement, HKB/VB of (*29*, *49*)] that is onlapped by the sedimentary drape (Figs. 2 and 3). Two regions of substantial volcanic relief are observed. VR1, located in the eastern corner of the NZ3D volume, contains at least two cones with ~1 to 3 km basal diameters and ~0.4 to 0.7 km vertical relief from the regional top of volcanic surface (Figs. 2B, 3, and 4B). VR2 is much more widespread throughout the NZ3D volume and contains topographic relief on at least two spatial scales. VR2 forms a broad ~10 to 15 km wide ridge that approximately follows the deformation front and connects to a subducted region of elevated relief near the northern edge of the volume (Figs. 2, A to C, 3, and 4B). At a smaller wavelength, VR2 consists of more than 15 volcanic edifices with both conical and anastomosing ridge morphologies. The small volcanic ridges are aligned with the general trend of VR2, implying that their feeder dikes were aligned by a common stress field or inherited crustal structure. Horizons at the base of individual volcanic edifices (e.g., VR2b and VR2c) demonstrate that some cones formed asynchronously, possibly as a field of monogenetic volcanoes (Fig. 2C). We observe cuspate reflectors with inclined edges and horizontal lengths of ~0.75 to 1 km beneath some of the cones (Fig. 2, B and C); we interpret these features to be frozen magmatic sills that intruded during the phase of volcanic edifice formation.

**Fig. 5.**
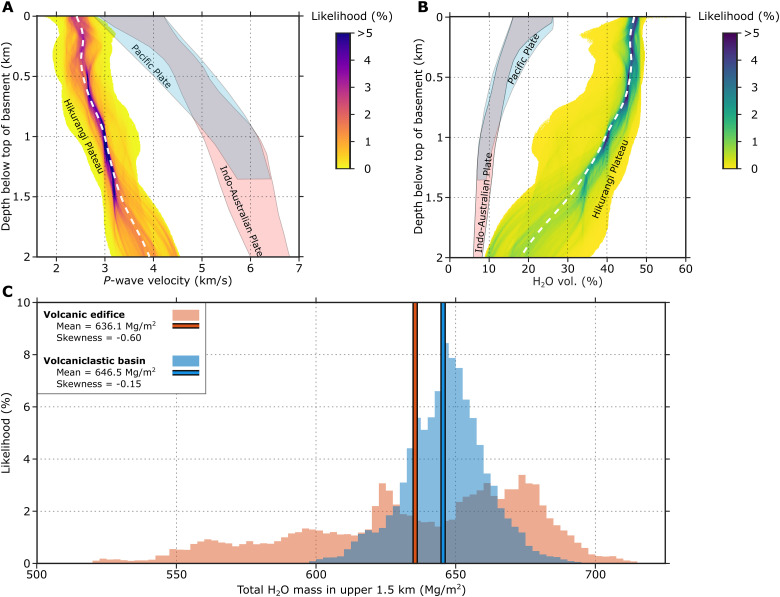
Seismically derived properties of the unsubducted Hikurangi Plateau upper crust. In all plots, likelihood is the histogram bin count normalized by the population size. (**A**) *P*-wave velocity histograms flattened to the top of volcanic upper crust. Bins are 0.01 km/s wide. Blue and pink polygons (overlap in gray) are ranges for normal oceanic crust of the Pacific and Indo-Australian Plates (*11*). The mean vertical function is shown as a white dashed line. (**B**) Volumetric water content histograms derived from *P*-wave velocity; formatting is identical to (A)—bins are 0.1 vol % H_2_O wide. (**C**) Total H_2_O mass, derived by converting vol % H_2_O in (B) to mass and integrating over the upper 1.5 km of each vertical function. Total H_2_O masses are segmented into volcanic edifice and volcaniclastic basin terrain populations. Bins are 0.250 Mg/m^2^ wide. Mean H_2_O masses are represented by bold vertical lines.

Distinct seismic facies within the volcanic upper crust indicate multiple formation mechanisms that may impart variations in rock properties. The top of the Hikurangi Plateau adjacent to the volcanic cone fields is a relatively smooth basin filled with ~0.2 to 0.4 km of volcaniclastic materials that thicken into the debris aprons of the volcanic regions (Figs. 2 and 4B). Wedges of laminated reflectors that emanate from the peaks of volcanic cones (Fig. 2B) imply pulses of eruption intensity and gravity flows that filled the intervening basin with highly fragmented, low-velocity volcaniclastic material (Fig. 2). In contrast, some cone flanks have chaotic, reflective debris aprons that could contain brecciated effusive materials and pillow lavas (Fig. 2C). Seismic velocities along the top of the volcanic upper crust seaward of the trench, including within the volcanic cones, are between ~1.8 and 2.9 km/s and exhibit the greatest variability within the volcanic cone field VR2 (Figs. 3 and 4C). *P*-wave velocities increase gradually with depth to ~3 to 4.5 km/s at ~1.5 to 2 km below the top of the Hikurangi Plateau (Fig. 5A), reaching values that are consistent with altered and fractured lavas at the top of basaltic oceanic crust (*11*, *59*, *60*). The low-velocity (<4 km/s) upper crust contains extensive subhorizontal intracrustal reflectors (ICRs) with lateral dimensions of ~1 to 5 km (Figs. 2 and 3). Based on their broad distribution beneath both the volcanic regions and volcaniclastic basins, we suggest that they originate from volcaniclastic and extrusive layers erupted onto the seafloor as the Hikurangi Plateau’s upper crust was constructed.

Our observations, including volcaniclastic aprons, intracrustal reflections, and widespread low velocities (<4 km/s), occur in an area much more extensive than the seamount edifices. They indicate that the upper ~1.5 km of the Hikurangi Plateau has a volcaniclastic origin, unlike normal basaltic oceanic crust. This layer acts as a thick upper crustal reservoir that stores abundant fluids in both pores and hydrated minerals. Volcanic materials can be strongly altered by low-temperature reactions with seawater such that solid components (e.g., olivine, volcanic glass, and plagioclase) are replaced by weaker fluid-rich clays (*61*, *62*). International Ocean Discovery Program (IODP) drilling results indicate that the upper Hikurangi Plateau contains large quantities of smectite-rich volcaniclastics, as well as other low-temperature hydrous phases such as zeolite (*29*, *56*, *57*).

**Fig. 6.**
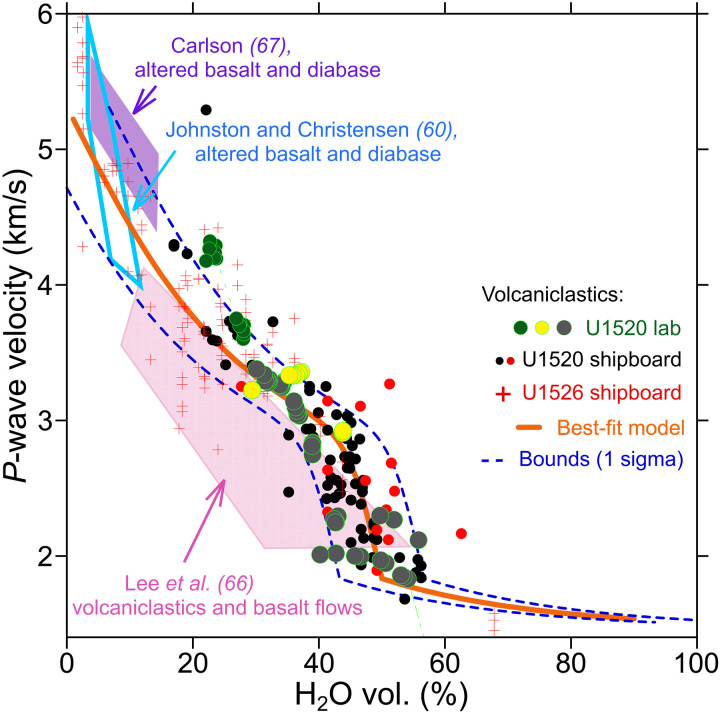
Compilation of *P*-wave and water content measurements on Hikurangi Plateau volcaniclastic samples from IODP Expedition 372/375 (*58*, *94*) and altered basaltic samples from other drilling expeditions. Site U1520 is located on the flank of Tūranganui Knoll, whereas U1526 drilled the seamount’s top. Data are well fit by an empirical relationship for siliciclastic sediments (*63*). Polygons highlight data ranges for samples of altered basalt and diabase (*60*, *67*) and volcaniclastic materials (*66*) from other ocean drilling studies. The orange line is the empirical relationship derived from Hikurangi Plateau volcaniclastics. Blue dashed lines represent plus or minus one standard error.

We aim to assess the fluid content of the Hikurangi Plateau volcaniclastic upper crust. It is important to consider both mineral and pore bound waters because their relative abundances determine the depths at which water can mobilize as a result of consolidation and dehydration. Porosity in marine sandstones with low clay content can be estimated from *P*-wave velocities when empirical relationships are calibrated with drilling data (*63*). However, this approach is not valid here because the smectite clay content of Hikurangi Plateau volcaniclastic samples often exceeds 50% normalized mineral abundance (*29*, *57*). Hydrated clay mineral phases have *P*-wave velocities that are slow relative to dehydrated clay mineral phases (*64*), and comparable to the nonhydrous phases with pore fluids present. Consequently, there is no reliable way to distinguish the abundance of pore and bound water in the subsurface solely on the basis of *P*-wave velocity (*65*). We estimate the total fluid volume from a relationship between total water content (bound and interstitial) and *V*_P_ using laboratory measurements on samples collected by drilling at sites U1520 and U1526 on IODP Expedition 372/375 (Fig. 1), augmented by published datasets for moderately altered basalt (Fig. 6) (*60*, *66*, *67*). The data are well-described by a relationship for porosity in sand-rich marine sediments (*63*), but for clay-rich sediments, they represent the total water content rather than porosity (Fig. 6). Our results show that the mean water content at the top of the incoming plate of the incoming Hikurangi Plateau upper crust is 47 ± 6.6 vol % H_2_O, and mean water content decreases to 31 ± 6.6 vol % H_2_O at 1.5 km depth in the Hikurangi Plateau (Fig. 5B). Our relatively high volumetric water content estimate is supported by independent estimates of high porosity (15 to 30%) reported from marine electromagnetics within the NZ3D survey area (*37*). This, along with high smectite clay abundances in x-ray diffraction data from site 1520 (*57*), suggests that up to half of water in the Hikurangi Plateau upper volcanic crust is mineral bound.

Whether rough volcanic topography directly delivers more fluid to the subduction zone is an important consideration. To address this, we vertically integrate water mass in the upper 1.5 km of the Hikurangi Plateau volcaniclastics at all inline/crossline pairs and segment inline/crossline pairs into populations of elevated (i.e., volcanic edifice) or low-lying (i.e., volcaniclastic basin) terrain (Fig. 5C). The volcanic edifice terrain distribution has nearly twice the range of the volcaniclastic basin terrain distribution and a stronger skew. The mean water masses of volcanic edifice and volcaniclastic basin terrains are 636.1 and 646.5 Mg/m^2^, respectively. The slight difference in water mass between the two populations may be due to faster basaltic intrusions within the volcanic edifices. Both the volcanic edifice and volcaniclastic basin regions are approximately 2.1 to 4.7 times more water-rich than the upper 1.5 km of normal oceanic crust (*11*), which we estimate contains between 151 and 246 Mg/m^2^ of water. We advise that this analysis compares the water contents of volcaniclastic units of the Hikurangi Plateau with lava and sheeted dike lithologies of normal oceanic crust. Thus, high-relief volcanic edifice regions contained within the NZ3D seismic volume have more heterogeneous water contents than volcaniclastic basin regions, but both regions contain far more water than normal oceanic crust.

### Hikurangi Plateau fluid response during subduction

**Fig. 7.**
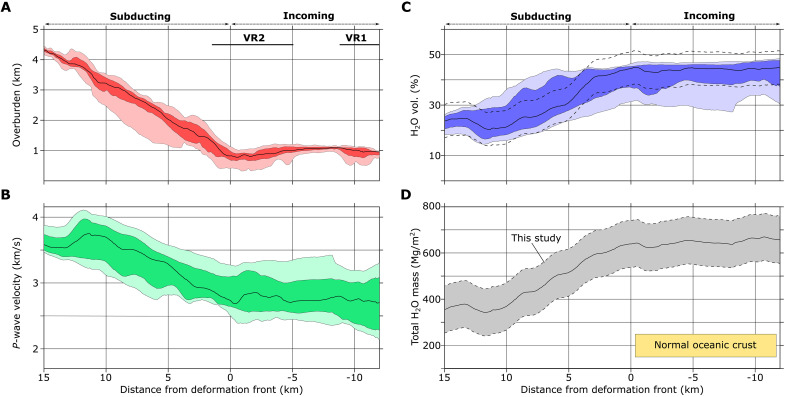
Variations in topography and volcanic upper crust properties binned by distance from the deformation front. In all plots, the black line indicates the median value, dark shading encompasses the interquartile range, and light shading encompasses the minimum and maximum (**A**) Overburden thickness obtained by subtracting the seafloor depth from the depth of the top of the Hikurangi Plateau volcanic crust. (**B**) Mean *P*-wave velocity vertical functions of the upper 1.5 km of the Hikurangi Plateau. (**C**) Volumetric water content determined from (B). Black dashed lines indicate one standard error of ±6.6 vol % H_2_O for the empirically derived *P*-wave velocity–water volume relationship. (**D**) Vertically integrated H_2_O mass per unit area of the upper 1.5 km of volcanic upper crust. Gray shading bounds the total H_2_O mass for one standard error. Yellow rectangle bounds the range of vertically integrated water mass in the upper 1.5 km of oceanic crust from (*11*).

The NZ3D volume contains clear evidence for fluid loss from the subducting upper volcaniclastic crust. Beneath the shallow megathrust within the 3D volume, the top of the Hikurangi Plateau has low relief, similar to the volcaniclastic basin between VR1 and VR2 (Fig. 4B). The northern extent of VR2 contributes to broad elevated topography near the northern corner within the study area and several subducted volcanic cones are observed within 10 km of the deformation front (Figs. 3 and 4B). In contrast, topography within the western corner of the study area is ~1 km lower and lacks resemblance to volcanic cone fields aside from a ~0.2 km tall knob. Upon subduction, seismic velocities within the upper 1.5 km of the Hikurangi Plateau increase with distance from the deformation front and the Hikurangi Plateau upper crust remains low-velocity compared to carbonate-rich hanging walls in the accretionary prism (*29*), forming a ~0.3 to 0.5 km thick low-velocity zone (LVZ) beneath the megathrust (Figs. 2A and 3). Within the subducting Hikurangi Plateau, the relatively low *P*-wave velocities (<3 km/s) persist until >8 km from the deformation front despite a substantial increase in the overburden and thus the total lithostatic stress (Figs. 2A, 3B, 4C, and 7, A and B). Within the most prominent LVZs, this corresponds to negligible water loss (~5 vol %) within the uppermost Hikurangi Plateau until the subducted crust has passed where the first major thrust fault intersects the megathrust (Figs. 4D and 7C). Remaining water content within the Hikurangi Plateau upon 15 km of subduction is between ~19 and 31 vol % H_2_O, equivalent to an average volumetric water loss of 14 to 26 vol % H_2_O (Fig. 7C). When integrated vertically in the upper 1.5 km, this corresponds to loss of ~300 Mg/m^2^ of water at 15 km beyond the deformation front, or ~20 Mg/m^2^ of water loss per kilometer of subduction (Fig. 7D).

The increase in *P*-wave velocities upon subduction is not uniform in the along-margin direction, reflecting 3D variations in dewatering (Figs. 3 and 4, C and D). Average seismic velocities within the top 1 km of the Hikurangi Plateau are >3 km/s between inlines 400 to 650 within ~2 km of the deformation front, whereas two broad LVZs (LVZ1 and LVZ2) persist to ~8 km from the deformation front at the northern and southern boundaries of the survey footprint (Figs. 3 and 4C). These along-strike differences in seismic velocity correspond to variations on the order of 10 to 15 vol % H_2_O (Fig. 4D). Upon subduction beyond 8 km, average velocities within the upper crust exceed 3.5 km/s within high-velocity zones (HVZs) that are separated by lower-velocity border regions (Fig. 4C). We find no obvious correlation to subducted topography and the velocity variations within the subducting plate. Both LVZs and the narrow HVZ are in equal proximity to VR2. Likewise, strong velocity variations appear unrelated to any relief in the region of relatively smooth topography on the subducting plate that is down-dip of VR2. Thus, we interpret the HVZs as regions with more efficient dewatering, corresponding to water losses on the order of ~20 to 25 vol % H_2_O (Fig. 4D). The intervening low-velocity regions have experienced less water loss (~15 vol % H_2_O). While some inherited variations in the proportions of pore- and mineral-bound water are possible, fault geometry in the overlying wedge has a substantial role. Thrust faults in the overlying plate and the décollement are the most likely candidates for channelized drainage pathways from the megathrust. LVZ2 lies directly beneath an accreted sediment lens that formed in the wake of a subducting seamount (*36*). Compared to the frontal wedge immediately south, the seamount increased the length of the Pāpaku fault and reduced the dip of both the Pāpaku fault and F1 in its wake (Fig. 4). This is believed to have created a less direct and presumably slower drainage pathway from LVZ2 (*36*).

## DISCUSSION

### A previously unknown fluid reservoir within Hikurangi Plateau volcanic upper crust

Recently acquired seismic data from the northern Hikurangi margin show that the subducting Hikurangi Plateau has a low-velocity ~1.5 km thick upper crustal layer rich in extensively altered and hydrated volcaniclastic sediment. Recent 2D studies of the northern Hikurangi Plateau in and near our study area confirm slow *P*-wave velocities (2 to 5 km/s) within a 2 to 3 km thick volcanic unit that overlies an additional ~1.5 km of upper crust (*31*, *68*, *69*) and correlates well with the thickness of electrically conductive basement (*37*). Our estimates place the average volumetric water content within this uppermost 1.5 km thick layer before subduction between ~32 and 47 ± 6.6 vol.%, with more fluid variation within volcanic edifices than in the more deeply buried flank basins (Figs. 5, B and C, and 7C). Despite its volcanic origin, this upper crustal layer is a large fluid reservoir that is equivalent to the fluid volume of many basinal sedimentary sequences at equivalent depths (*63*, *65*).

Previous studies show that basaltic oceanic crust of seafloor spreading origin typically has small fluid content compared to marine sediments. Water storage within mature basaltic upper oceanic crust includes pore-bound water (typically <5 to 10 vol % H_2_O) and mineral-bound water, primarily within clays and opal (*61*, *67*). Average clay abundance in basaltic upper oceanic crust is not well documented globally, but sparse local measurements show that it can vary widely, between 0 and 15 wt %, and highly altered cracks or hyaloclastites can exceed 20 wt % but are generally of limited spatial extent (*62*, *70*). Slow *P*-wave velocities (2 to 4.5 km/s) in young upper oceanic crust (*59*, *71*, *72*) are attributed to high initial porosity (*73*), but the seismic velocity structure of basaltic upper oceanic crust follows predictable aging trends that result in faster seismic velocities. Global compilations show that *P*-wave velocities at the top of old oceanic crust (>7.5 Ma) gradually increase with age from 4.5 to 5 km/s (*59*). High-resolution seismic tomography studies confirm that mature basaltic upper crustal velocities tend to increase from ~3 to ~6 km/s with depth in the upper 1.5 km that correspond to water contents of 5 to 25 vol % H_2_O (*11*, *71*) (Fig. 5, A and B). In typical oceanic crust, these depth ranges (i.e., 0 to 1.5 km below top of basement) encompass both extrusive volcanics and sheeted dike intrusives, the latter of which have much lower porosities on the order of ~4% (*74*). The northern Hikurangi Plateau’s upper crustal velocities (1.8 to 3.9 km/s) are unusually slow relative to global averages for the top of Cretaceous-age oceanic crust (~5 km/s) (*59*), and its range of estimated presubduction water contents (17 to 50 vol % H_2_O) greatly exceeds global porosity trends within Cretaceous upper oceanic crust (5 to 11%) (*61*). In terms of total vertically integrated water mass, the upper 1.5 km of the northern Hikurangi Plateau volcanic basement holds ~520 to 750 Mg/m^2^ of water before subduction (Figs. 5C and 7D), a mass ~2.1 to 4.7 times greater than upper basaltic oceanic crust water masses from the Pacific Plate offshore Alaska and the Indo-Australian Plate at Sumatra where good estimates exist (*11*).

Plate bending normal faults between the outer-rise and trench are responsible for hydrating oceanic crust and lithospheric mantle outboard of many subduction zones worldwide (*11*–*15*). In the NZ3D volume, we do not observe evidence of active basement normal faults within the Hikurangi Plateau before subduction that would be expected to clearly offset the top of the Hikurangi Plateau upper crust and overlying sediments, in contrast with prior interpretations (*29*, *75*). Thus, the fluids and faults within the unsubducted Hikurangi Plateau are likely the result of ancient hydrogeologic and deformation processes that accumulated fluid reservoirs before substantial sediment cover was deposited.

Oceanic LIP crust is generally understood to be ~1.5 to 4 times thicker than normal oceanic crust (*76*), but has not previously been considered as a major source of subducting fluids. If LIP upper crust is thickened or formed by a different mechanism, its fluid storage capacity could be enhanced beyond that of oceanic crust formed at seafloor spreading ridges. Our understanding of LIP upper crust is limited to shallow drilling and seismic profiling that indicates voluminous eruptions that in some cases occur in shallow water (<1 km) and form porous volcaniclastic crust (*66*, *76*, *77*). We observe only a slight difference in the total fluid storage capacity within the study region regardless of whether the upper crust is a volcaniclastic basin or volcanic edifice (Fig. 5C), suggesting that the mechanisms that produced high porosity and clay content in volcaniclastics were widespread. Sites of volatile-rich shallow water eruptions (*78*) or volcanic debris fans (*79*) could have higher inherent porosity than intact pillow basalts that would enhance alteration. In addition, greater abundances of materials that alter to smectite, such as volcanic glass, olivine, and plagioclase, could increase the extent of hydration.

The prolonged magmatic evolution of the Hikurangi Plateau, with at least two major phases of volcanism in the Early and Late Cretaceous and an intervening period of rifting from the Manihiki Plateau along the Rapuhia Scarp, as well as Late Cretaceous subduction at the Chatham Rise (*49*, *51*), presents the possibility of varying environmental and magmatic conditions that could affect upper crustal hydration. Geologic conditions at the earliest phase of Hikurangi Plateau formation that coincide with the Greater Ontong Java event are poorly constrained and unsampled by drilling. However, there is evidence of shallow marine and subaerial eruptions at the Ontong Java Plateau in the form of accretionary lapilli bearing pyroclastic rocks (*80*). Drilling within the Manihiki Plateau recovered highly vesicular basalts that erupted at water depths shallower than 400 m, implying ~3 km of postmagmatic subsidence (*81*). Wave-cut terraces and conglomerates from tidal environments on nearby Hikurangi Plateau seamounts indicate that relative sea level was at least ~2.8 km lower during the second magmatic phase in the Late Cretaceous (*51*), which could provide conditions that allow for more vesicle formation and explosive fragmentation of submarine tephra.

It is not clear whether variations in fluid storage capacity exist across the Hikurangi Plateau that could contribute to margin parallel variations in fluid delivery to the Hikurangi megathrust. The limited spatial extent of the NZ3D survey prevents us from investigating broader changes in the upper crustal structure across the Hikurangi Plateau. However, there is reason to suspect that past tectonic events and inherited volcanic deposits could cause substantial variations. Layered reflections within the Manihiki Plateau upper crust, similar to the ICRs reported here (Figs. 2 and 3), correspond to volcaniclastic and basaltic units that reach local thicknesses of several kilometers and vary with proximity to volcanic eruption centers (*82*). The northernmost Hikurangi Plateau rifted from the southern Manihiki Plateau in the Early Cretaceous (*48*, *49*). Multichannel seismic and gravity data demonstrate that the northern Hikurangi Plateau is a ~250 to 300 km wide region of high and rough volcanic topography compared to the plateau further south (*49*). The plateau rifting process may have resulted in broadly distributed volcanism and deformation across the northern Hikurangi Plateau that hydrated the upper basement and contributed to the alignment of volcanic edifices (Figs. 1 and 4). To the south, the Hikurangi Plateau was further from the site of rifting and subducted beneath the Chatham Rise in the Late Cretaceous. Ancient subduction and deep burial along the Chatham Rise could have resulted in an initial phase of dewatering that did not occur further north (*83*). Coarser seismic profiling of the southern Hikurangi Plateau suggests faster upper crustal velocities compared to the NZ3D volume (~4.5 to 5.2 km/s) (*54*), which implies rocks with less fluid content, but this remains to be confirmed by high-resolution seismic tomography and drilling.

Some have proposed that large seamounts are responsible for transporting large volumes of fluids to the subduction system (*38*) that may ultimately influence fault slip behavior, and play a role in shallow slow slip events (*37*). Our results and nearby electromagnetic studies (*37*) show that the upper 1.5 km of the northern Hikurangi Plateau’s volcanic crust is water rich, regardless of whether it formed as a distributed volcanic field, or an adjacent volcaniclastic basin. Whether subduction of large seamounts or volcaniclastics results in different dewatering processes is an open question. The nearby seamount, Puke Knoll, contains slightly faster material (3 to 4 km/s) within its upper 1.5 km (*31*) that suggests lower volumetric fluid content in the upper crust of large seamounts, presumably with stronger, more magmatically intruded interiors than their volcaniclastic flank aprons. This is also supported by the seismically faster, more electrically resistive, and less water-rich basaltic cap lavas drilled on top of the nearby seamount Tūranganui Knoll compared to its more water-rich volcaniclastic flank (Fig. 6) (*29*, *37*). While this may suggest that large seamounts are less fluid rich than their volcaniclastic flank basins, seamounts should also be viewed as regions with locally thickened upper crust and enhanced hydrothermal fluid flow that could have higher average porosity and alteration than normal oceanic crust (*37*–*39*). Three to four kilometers of thickened volcanic basement with ~9 to 13% porosity, as reported from electromagnetic methods at Tūranganui Knoll, would deliver vertically integrated water masses on the order of 278 to 536 Mg/m^2^. This water mass should be considered an underestimate because electrical resistivity can be more sensitive to pore water than mineral-bound water (*37*). Thus, both the volcaniclastic basement and large seamounts of the Hikurangi Plateau contain substantially more water than normal oceanic crust. However, large seamounts and the volcaniclastic basement unit could differ in how they release fluids upon subduction if they have different (i) proportions of pore- and mineral-water (ii) transport length scales and permeabilities, (iii) thermal conditions, or (iv) rock matrix compressibilities.

### Influence on subduction hydrogeology and slow slip

The unusually thick extent of low-velocity volcanic upper crust of the northern Hikurangi Plateau provides conditions that allow for large-scale fluid mobilization from the subducting plate. Porosity reduction upon subduction would also reduce the permeability of the uppermost Hikurangi Plateau, increasing the tendency for excess pore-pressure generation down-dip with further progressive loading and the onset of substantial low-temperature clay mineral dehydration (*1*). Because the Hikurangi subduction zone is relatively cold (*24*, *84*), mineral dehydration in the basaltic upper oceanic crust within ~15 km of the trench should be limited, and occur only in the lowermost part of the thick volcaniclastic section where temperatures reach ~80° to 100°C. Thus, much of the ~15 to 20 vol % H_2_O reduction in water content by 15 km from the deformation front probably reflects porosity loss (Figs. 4D and 7C). Fluids escaping the subducted volcanic upper crust could flow laterally through the subducting plate, exit upward and along the megathrust, or across the megathrust into splay faults. The latter scenario is supported by the delay in fluid loss until subducting volcanic materials reach the point where the first thrust fault intersects the megathrust (Fig. 4D). Sparse thermal measurements on the incoming plate suggest that lateral fluid transport through the Hikurangi Plateau outboard of the deformation front is limited (*84*) and drilling through the Pāpaku fault showed no evidence of recent flow (*85*). The drainage process is not uniform in three dimensions. Given the comparably low variation in seismic velocity and water content in the Hikurangi Plateau before subduction, we speculate that the along-strike variations in these properties of the subducting Hikurangi Plateau (Fig. 4C) show that dewatering is influenced by 3D variations in permeability and loading. This includes the possibility that hydrogeology of the upper plate influences drainage from the Hikurangi Plateau. Large lenses of poorly drained sediments are prevalent in the upper plate within the NZ3D volume (*36*), the most shallow of which directly overlies LVZ2. Limited drainage of the upper plate will necessarily lead to excess pore fluid pressures within the underthrust volcanic crust if fluids drain through upper plate faults and formations.

Here, dewatering of subducting crust is in direct contrast to many other subduction zones, such as the Middle America Trench, where drilling results and pore water geochemical data suggest that the top of the subducting oceanic crust represents a distinct hydrologic system that dewaters at greater depths, independent from the fluid system of the subducting sediments, megathrust, and upper plate (*8*, *41*). Basaltic oceanic crust is typically much stronger and less porous than marine sediments such that vertical loading of basaltic oceanic crust upon subduction fails to drive compaction or fluid drainage in near-trench hydrogeologic models (*43*). Additionally, low-permeability pelagic deposits that typically immediately overlie the igneous basement have been invoked as a barrier that separates flow within the permeable upper igneous crust from that in the overlying sedimentary section (*86*). For the case along the northern Hikurangi margin, fluid mobility from the subducting crust is much higher and substantial drainage must occur through the forearc where we propose that volcaniclastic-rich lithologies contain a large reservoir of water that can be expelled by consolidation.

The properties of the Hikurangi Plateau have key implications for predicting fluid transport and stress state within the Hikurangi subduction zone. The clear LVZ within the upper crust of the subducting plate (Figs. 2A, 3, and 4C) is unique compared to other high-resolution tomographic studies of shallow subduction (*87*) and indicates that accreted sediments within the upper plate are elastically stronger than the subducting volcanic upper crust. This configuration is similar to LVZs within subducting sediments at the southern Hikurangi margin (*88*) and other margins that sometimes reflect poor drainage, low effective normal stresses, and elevated pore fluid pressure (*25*, *26*), but with volcaniclastic materials instead of more typical marine sediments. Low *P*-wave velocities within the subducting volcanic upper crust could be caused by delayed fluid drainage in response to vertical loading or an excess of fluid supply from down-dip. However, our results also confirm that fluids are draining from the Hikurangi Plateau upper crust and LVZs could be partially the result of lithology differences between the upper carbonate-rich and lower volcaniclastic-rich plates rather than only the stress state.

Low effective normal stress as a result of elevated pore fluid pressure influences frictional stability and produces conditions that should promote slow slip (*22*, *23*). We cannot accurately estimate pore fluid pressure within the Hikurangi Plateau upper crust because well-established compaction curves do not exist for volcaniclastic lithologies, and because the velocity data do not allow us to distinguish between pore- and mineral-bound components. However, several observations in addition to the LVZs in the subducting plate suggest that elevated pore fluid pressure is possible despite the observation of dewatering: (i) reduced permeability in response to partial drainage (*9*), high clay content, substantial remaining fluid reservoirs, and the large total thickness of the Hikurangi Plateau volcaniclastic unit would promote elevated pore fluid pressure generation as subduction continues beyond our region of interest (*1*); (ii) dynamic triggering of a large slow slip event in response to the 2016 Kaikoura earthquake (*89*) is consistent with low effective normal stress (~0.4 MPa) along the northern Hikurangi megathrust (*90*); and (iii) episodic stress cycling inferred from focal mechanism inversions indicates that fluid pressures vary temporally within the Hikurangi Plateau and peak near lithostatic at the onset of slow slip events (*91*). The latter observation and the lack of notable subducting sediments fluid sources suggests that the Hikurangi Plateau is a source of fluids that is hydraulically linked to the megathrust and influences slow slip. The Hikurangi Plateau also subducts in the locked southern Hikurangi margin, which lacks frequent shallow slow slip. However, there, smoother crust and laterally continuous subducting siliciclastic sediments could hydraulically isolate the subducting basement from the megathrust (*28*, *88*), in addition to the possibility of inherited differences of upper crustal hydration between the northern and southern Hikurangi Plateau.

In conclusion, we argue for a more expansive view regarding the role of intraplate volcanism in transporting fluids to the subduction system: Regions with thickened extrusive and volcaniclastic crust have amplified fluid reservoirs that can influence forearc hydrogeology if they are hydraulically connected to the megathrust. Water content (~18 to 30 vol % H_2_O) at the deepest subducted edge of our study remains in excess of expected fluid volumes within unsubducted upper basaltic oceanic crust [6 to 26 vol % H_2_O; (*11*, *12*)], which implies ample fluid delivery to slow slip source regions. Geochemical signatures from onshore seeps in the Hikurangi forearc and volcanoes in the southern Kermadec arc and Havre trough system also indicate deeper fluid fluxes from the Hikurangi Plateau through the upper plate, implying that the large fluid sources within the Hikurangi Plateau persist to depths greater than shallow slow slip (*3*, *53*, *92*). Because few subduction zones have subducting sediment layers >1 km (*93*), our results imply that the northern Hikurangi margin can have a fluid budget as large or larger than most subduction zones despite its lack of subducting marine sediment. Regions with thin subducting sediments, and thickened volcanic upper crust, including the Cocos Ridge (*21*, *32*), the northern Ryukyu trench (*33*, *34*), and other subducting volcanic provinces (*27*), may exhibit excess fluid inputs from the subducting volcanic upper crust that influence forearc hydrogeology and shallow slow slip.

## MATERIALS AND METHODS

### NZ3D seismic data

In 2018, *R/V Langseth* (cruise MGL1801) acquired a 15 × 60 km^2^ area controlled-source seismic dataset using two 3300 in^3^ tuned source arrays, each consisting of 18 airguns towed at 7 m depth with 75 m lateral separation firing in flip-flop mode. The total seismic survey included 62 passes with a total length of 5489 km and 143,078 shots. Returning seismic energy was recorded on a towed receiver array comprising four 6 km, 468-channel solid-state hydrophone streamers. The hydrophone streamers were separated in the crossline direction by 150 m and towed at 8 m beneath the sea surface. Shots were simultaneously recorded on 97 JAMSTEC short-period ocean bottom seismometers (OBS) placed at ~2 km separation within the survey area by *RV Tangaroa* (cruises TAN1712 and TAN1803). The seafloor receivers were equipped with three orthogonal seismic channels and a hydrophone.

The seismic data volume was processed by the commercial contractor CGG Services (Singapore) Pte. Ltd. in 2020–2021 to obtain consistent seismic reflection and *P*-wave velocity volumes with minimal noise (*36*, *47*). Detailed sequential data processing descriptions and testing can be found within the extensive data technical reports published with the open-access datasets (*45*, *46*). Key elements of data preprocessing include 3D deghost and designature, model-based water demultiple with water bottom modeling (MWD), 3D iterative surface-related multiple attenuation (SRMA), 2D Radon demultiple, and 3D binning and data regularization. *P*-wave velocity model building was performed with joint acoustic full-waveform inversion and reflection tomography with tilted-transverse isotropy (TTI). First iterations of full waveform inversion (FWI) were isotropic and performed using 3D streamer data at 5 Hz. Subsequent FWI iterations included seafloor hydrophone channels up to 25 km offset, TTI FWI up to 12 Hz, low Q compensation for an anomalous zone of attenuating gas below the bottom simulating reflector, and TTI reflection tomography to refine deep plate boundary structure. Velocity model performance was assessed by misfit of waveforms in FWI and flattening of common image gathers (CIGs) through iterations of PSDM. Final velocity model perturbations within the Hikurangi Plateau volcanic upper crust are on the order of ±0.15 km/s, which is ~4 to 7% of the unit’s *P*-wave velocity (2 to 4 km/s). The region of interest in this study, which includes the upper crust of the Hikurangi Plateau within 15 km of the trench, is well coved by turning rays recorded on OBSs and by reflections recovered on both the OBSs and hydrophone streamer to 8 km depth on the incoming plate and beneath the outer prism (*36*, *47*). For the dominant FWI frequencies used (5 to 12 Hz), the maximum achievable resolution, assuming sufficient illumination and receiver geometry, is half the dominant wavelength, which is ~83 to 200 m at 2 km/s and ~166 to 400 m at 4 km/s. However, the ~2 km average instrument spacing of OBSs will result in lower-velocity resolution than these upper bounds, particularly in the deeper volcanic section. The final seismic imaging step applied 3D TTI PSDM with 5 km half aperture, 18.75 m inline spacing, and 12.5 m crossline spacing.

### Top of Hikurangi Plateau mapping

Horizon mapping was performed with Paradigm seismic interpretation software packages (http://www.pdgm.com). The top of the volcanic Hikurangi Plateau was identified as a regional unconformity between volcanic cones and adjacent basins, and their overlying sediments (*29*, *49*). Horizon picks were selected manually on inline at intervals of five inlines (93.75 m), and intersecting crosslines were simultaneously viewed to ensure consistency. Correlation thresholds (~70 to 90%) were used to auto-track between inline picks where the unconformity could be followed successfully. Picks were then interpolated to a 3D grid. The top of the volcanic Hikurangi Plateau horizon was filtered with a 5 × 5 element moving average window (75 m crossline direction by 50 m inline direction).

### Relationship between volcaniclastic *P*-wave velocity and water content

Cores and well-logs of the crust of oceanic LIPs are rare and restricted in depth, limiting our ability to accurately apply relationships that estimate rock properties (e.g., porosity and clay content) from *P*-wave velocity. However, IODP Expedition 372/375 drilled >200 m into the upper volcanic units of the Hikurangi Plateau at sites U1520 (*58*) and U1526 (*94*), ~2 and ~10 km to the southeast of the NZ3D survey, respectively. Site U1520, located on the volcaniclastic apron of Tūranganui Knoll, recovered ~197 m of volcaniclastic conglomerates with interbeds of marl and silty claystone above basalt. Site U1526 sampled 51 m of coarse volcaniclastic sandstones, vesicular basalt, and volcaniclastic conglomerate at the of Tūranganui Knoll. Substantial excursions from mean physical properties (i.e., porosity and *P*-wave velocity) occur within the volcanic units at both sites, reflecting sharp variations in pore-filling minerals, clay alteration (up to 95 vol %), primary mineralogy, and porosity (*29*).

Clay minerals hold similar volumes of water in basaltic upper oceanic crust compared to the pore volume that they displace (*61*), and hydrated smectite mineral *P*-wave velocities are nearly the same as the velocity of water (1.6 and 1.5 km/s, respectively). Therefore, we derive an empirical relationship between *P*-wave velocity (*V*_P_) and volumetric water content based on laboratory IODP shipboard core measurements at sites U1520 and U1526 by fitting these data to a commonly used empirical relationship for marine sediments (*63*):

$$V_{P}=1.11+0.178\phi +\frac{0.305}{{\left(\phi +0.135\right)}^{2}+0.0775}+0.61(v_{\mathrm{sh}}-1)\chi$$

$$\chi =\tanh(20[\phi -\phi_{c}])\;-|\tanh(20[\phi -\phi_{c}])|$$

where ϕ is volumetric water content including both interstitial and mineral-bound water, *v*_sh_ is a fitting parameter (0.16854), and ϕ_c_ is critical volumetric water content (0.499). An expanded description of experimental procedures is provided in the Supplementary Materials. The standard error of this fit between water content and *V*_P_ is 6.6 vol.% water. We use a grid search to solve for volumetric water content from *V*_P_. To estimate vertically integrated water mass per unit area, we multiply vertical volumetric water content or porosity functions by the density of seawater (1030 kg/m^3^) and integrate over a depth range using trapezoidal numerical integration.
