# Supplementary material for: Subducting volcaniclastic-rich upper crust supplies fluids for shallow megathrust and slow slip
Source: Sci Adv. 2023 Aug 16;9(33):eadh0150. doi: 10.1126/sciadv.adh0150 (PMC10431706; doi:10.1126/sciadv.adh0150)
Supplement: Supplementary file 1 — Supplementary Text Fig. S1 [file sciadv.adh0150_sm.pdf]

Science Advances
AAAS

Supplementary Materials for

Subducting volcaniclastic-rich upper crust supplies fluids for shallow megathrust and slow slip

Andrew C. Gase *et al.*

Corresponding author: Andrew C. Gase, gasea@wwu.edu

Sci. Adv. **9**, eadh0150 (2023)
DOI: 10.1126/sciadv.adh0150

This PDF file includes:

Supplementary Text
Fig. S1

Supplementary Text

Description of Laboratory Procedures

We conducted normal consolidation experiments on sediment cores collected from site U1520 by IODP expedition 372/375 at room temperature. Total water content of the samples was determined directly. The wet and dry masses were measured, and dry grain density was determined with a pycnometer. All samples were trimmed parallel to the whole round cores to a diameter of 25.4 mm and a length between 15 and 20 mm. Once trimmed, the samples were placed in the uniaxial apparatus under a normal load of 350 kPa and a pore-pressure of 300 kPa and left to equilibrate for ~24h before commencing the constant rate-of-displacement ramp. The normal load was applied via a computer-controlled screw-driven stage and axial displacement was monitored using a linearly variable displacement transducer (LVDT). We applied the axial load by setting a constant displacement rate of 0.005 mm/min to 0.015 mm/min. We chose the displacement rates to keep the pore-pressure equal to or less than 15% of the applied normal stress. The fluid pressure at the top of the sample was held constant at 300 kPa while the base of the sample was closed prevent drainage. We monitored the fluid pressure in the undrained base throughout the test with a pore-pressure transducer.

We collected acoustic waveforms campaign style along the deformation path using the time-of-flight technique, in which we applied an excitation to a lead-zirconate transducer (PZT) with a center frequency of 500 kHz and received the arriving signal downstream with an identical PZT. The waveforms were sampled at 50 MHz and the P-wave arrivals were picked manually. P-wave arrival times were corrected empirically for flight time through the steel end-platens and the porous metal frits. Additionally, the measured sample displacement was also corrected empirically for deflection of the apparatus.

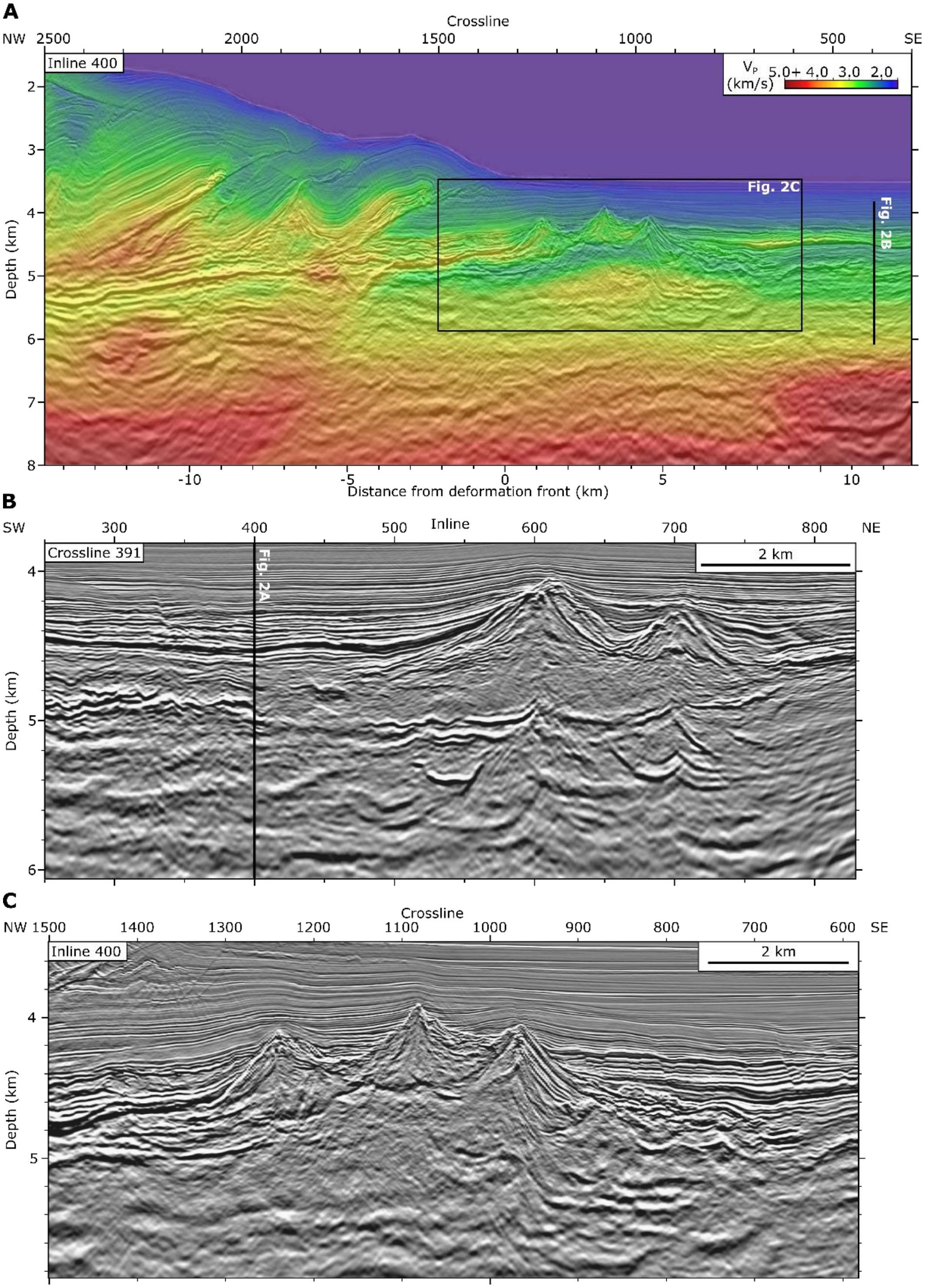

Fig. S1. Uninterpreted seismic panels. (A) Segment of Inline 400 with P-wave velocity overlain. **(B)** Segment of crossline 391. **(C)** Zoom in of Inline 400 from **(A).**
